# Aesthetic Reconstruction Based on Facial Subunit Principle for Basal Cell Carcinoma of the Face: A Retrospective Analysis

**DOI:** 10.7759/cureus.56826

**Published:** 2024-03-24

**Authors:** Biswajit Mishra, Surya Mallik, Ishan Agnihotry, Jibitesh Behera

**Affiliations:** 1 Department of Plastic Surgery, SCB Medical College, Cuttack, IND

**Keywords:** scar management, surgical oncology, dermatologic surgery, basal cell carcinoma, facial subunit, aesthetic reconstruction

## Abstract

Background and objective

Basal cell carcinoma (BCC) is the most common malignancy of the skin. Reconstruction of post-excisional defects in BCC should follow the subunit principle for better outcomes. The location of BCC of the face is determined based on facial units; however, very few studies have described the involvement of multiple units and multiple subunits in BCC. In this study, we aimed to provide valuable insights into the management of BCC involving various facial units and subunits, thereby contributing to improved patient care and outcomes.

Materials and methods

We conducted a retrospective study at the Plastic Surgery Department of the SCB Medical College in Cuttack, Odisha, from January 2020 to January 2022, after obtaining ethical approval from the SCB Medical College IRB (no: 1155). We examined 35 patients with BCC of the face. The inclusion criteria were as follows: patients with early-stage and primary tumors that were mobile, not attached to underlying bone or cartilage, and amenable to surgical resection. Conversely, patients with late-stage, neglected, and recurrent tumors, fixed tumors, or those infiltrating the underlying bone or cartilage were excluded from the study. Data collection involved retrieving pertinent information from medical records, including parameters such as age, sex, tumor site, type of flap utilized, follow-up, and any complications observed. The tumor sites were further divided into six separate groups based on facial aesthetic units: the forehead, the nose, the area around the eyes, the cheek, the mouth, and the area around the ear, each with its own subunits.

Results

A total of 35 patients were included in this study, comprising 15 males (42.85%) and 20 females (57.15%), with a male-to-female ratio of 1:1.33. The ages of the patients ranged from 42 to 68 years. Among the facial units, the nose was the most commonly involved (in seven cases), while the lip was the least commonly affected (in one case). In 24 cases, a single unit was involved, while 11 cases involved multiple units. Furthermore, single subunits were affected in 18 cases, double subunits in 10 cases, three subunits in five cases, four subunits in one case, and five subunits in another case. Notably, no cases exhibited flap necrosis, wound dehiscence, wound hematoma, or seroma, indicating excellent surgical outcomes. All flaps remained viable, and all patients were followed up for a minimum of one year, with no reported recurrence during the follow-up period ranging from 6 to 18 months, reaffirming the effectiveness of the treatment approach.

Conclusions

For small, superficial lesions, full-thickness skin grafts (FTSG) are a suitable treatment option. However, when dealing with larger lesions that encompass multiple subunits, the preferred approach involves reconstructing with locoregional flaps. It is essential to plan the procedure carefully, taking into account the goal of positioning the final scar along the junction of facial subunits. This strategic plan aims to achieve superior aesthetic outcomes.

## Introduction

Surgical excision is the most commonly performed procedure for primary basal cell carcinoma (BCC) and has become the standard treatment method where Mohs micrographic surgery is not available. A 3-7 mm excision margin provides a negative margin in the majority of cases [1]. Reconstruction with local flaps rather than direct closure provides better wound closure in BCC on the face, particularly in medium-sized and large defects [1]. Principles of facial cosmetic units should be followed to achieve optimal results during the reconstruction of post-excisional facial defects [2]. The initial concepts of facial reconstruction were proposed by Gonzales-Ulloa in 1956 [2], who emphasized the need to put an end to the “age of skin patch surgery” and proposed a method of selective regional restoration using “aesthetic units.” This method consists of making cutaneous grafts of the same size, shape, and thickness as the whole region on which the repair is done.

Each facial subunit is made up of areas that are bounded by natural folds and borders, like the nasolabial fold, mental crease, vermilion, brow, and hairline. The skin color, texture, and thickness are mostly the same, as well as the amount of subcutaneous fat, mobility, and hair distribution. The main cosmetic units are, in turn, subdivided into various subunits [3]. Post-excisional defects should also follow the principles laid down by Millard in 1981 [3], which emphasize that reconstruction should be based on facial features, including the three basic layers: cover, frame, and lining. In 1985, Burget and Menick [4] established the initial concepts of aesthetic subunits in nasal reconstruction. Since then, these concepts have been considered important steps in preoperative planning.

The face is typically divided into six units: forehead, eye and eyebrow, nose, lips, cheek, and chin. Each of the units has been further subdivided into various subunits by Russo [5-11]. In the literature, the location of BCC is predominantly described based on facial units, but very few studies have described the involvement of multiple units and multiple subunits in BCC. Though Russo has described flaps of choice for single subunits, there is no clear-cut guideline for flaps involving multiple subunits. This study focuses on categorizing BCC locations into different groups depending on their impact on various facial units and subunits. It also explores the application of subunit repair principles in reconstructing individual units and multiple units.

## Materials and methods

Study design and setting

This was a retrospective study involving 35 patients with BCC of the face (BCC) who were admitted to the Plastic Surgery Department, SCB Medical College, Cuttack, Odisha, from January 2020 to January 2022 (two-year duration). The inclusion criteria were as follows: patients with early-stage and primary tumors that were mobile and not attached to the underlying bone or cartilage and were curable by surgical resection. Exclusion criteria included patients with late-stage, neglected, and recurrent tumors, fixed tumors, or those infiltrating the underlying bone or cartilage and were not curable by surgical resection.

Data collection

Data were collected from patient medical records and included the following parameters: age, sex, site of the tumor, type of flap, follow-up, and complications. The site of tumors was divided into six groups based on the principle of facial aesthetic units (forehead, nasal, periorbital, cheek, perioral, and periauricular) and their subunits.

**Figure 1:**
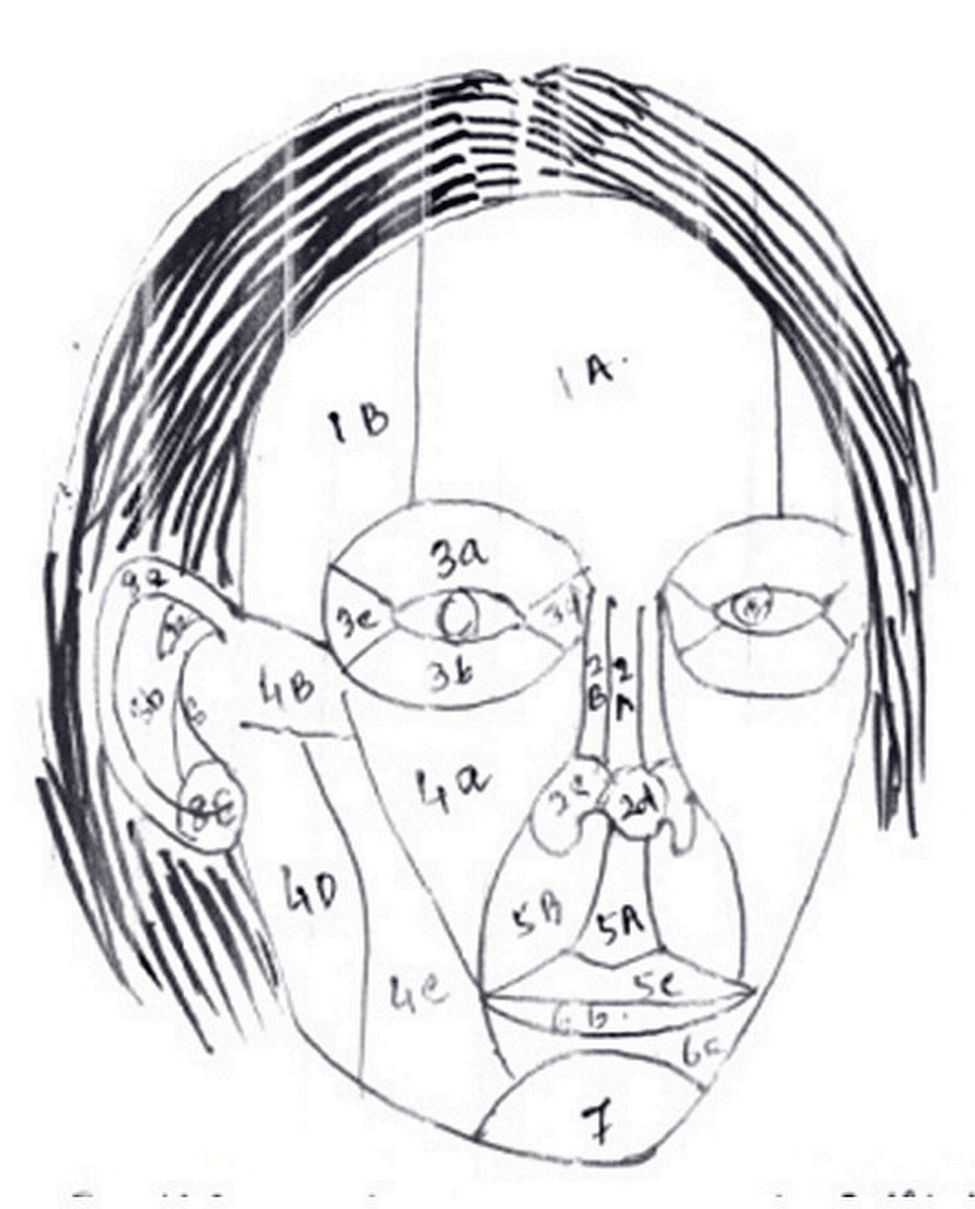
Reference image depicting the subsections on the face

Each unit and subunit was numbered as described by Russo [5]. The basic concept is that there is a difference between a unit and subunits. There are nine major units in the face (scalp, forehead, eyelid, nose, upper lip, lower lip, chin, cheek, and ear). Each unit is further classified into various subunits by Russo, amounting to a total of 27. First, we tried to classify the lesion according to unit involvement. The nose was the most common unit (seven cases), and the lip was the least common unit (one case). On further analysis, it was found that the lesion had not been confined to a single unit, and it had involved adjacent units as well. Hence, we tried to classify accordingly, which we have termed multiple unit involvement (Figure 1).

There was a single isolated forehead defect affecting the 4a subunit. In seven cases, the lesion was confined to the nasal units. The distribution of subunits affected in these cases is as follows: 2a (dorsum of the nose) in two cases; 2a and 2b (dorsum and lateral nasal wall) in one case; 2b and 2c (lateral wall and ala) in one case; 2d (tip) in two cases; and 2b, 2c, and 2d (lateral wall, ala, and tip) in one case. In some instances, the nose was involved along with adjacent units. In one case, a double subunit was affected (2a and 3d: the dorsum of the nose and medial canthus). Three subunits were involved in one case (2b, 2c, and 4a: lateral wall and ala of the nose and cheek). In another case, four subunits were affected (2a, 2b, 3d, and 4a: the dorsum, lateral wall, tip of the nose, and medial canthus of the eye). There was also a case with five subunits involved (2a, 2b, 3b, 3d, and 4a). Reconstruction was performed using both flaps and grafts. Local flaps were the primary choice in all cases. These local flaps were categorized as random pattern flaps or axial pattern flaps, depending on their vascular supply. Random pattern flaps were employed in 14 cases, with the Limberg flap being the most frequently used (four cases), followed by the glabellar flap (three cases), the bilobed flap (one case), the local transposition flap (three cases), and the cervicofacial flap (three cases). Axial-pattern flaps were utilized in 13 cases. Among these, the forehead flap was used in five cases, the nasolabial flap in three cases, the Mustardé flap in four cases, and the dorsal nasal flap in one case. In addition to flaps, full-thickness skin grafts (FTSG) were employed in seven cases, while a composite graft was used in one case.

Various flaps were utilized for nasal reconstruction, including one Rieger flap, three glabellar flaps, one bilobed flap, five forehead flaps, and one nasolabial flap. The forehead flap was commonly chosen for cases involving multiple subunits of the nose, such as the tip and ala. It was also used when extensive reconstruction was required, such as when the nose was involved along with the medial canthus, cheek, or eyelid units. The nasolabial flap was preferred for smaller areas of the ala or tip.

Example case

A 59-year-old female presented to our outpatient department with a previously diagnosed BCC affecting the ala, lateral wall of the nose, and cheek region. Post-excision, a complex defect remained, involving full-thickness loss of a portion of the nose and cheek, corresponding to the 2b, 2c, and 4a subunits of the nose. A paramedian forehead flap was designed for the reconstruction. The flap's pedicle was formed by an anastomosing branch between the angular artery and supratrochlear artery, extending from the brow to the hairline. The flap was designed vertically and axially, with a narrow width at the pivot point (approximately 1.5 cm). It was raised from the distal to the proximal direction. In the proximal one-third, the flap was narrow, and in the distal two-thirds, it expanded. The flap was dissected above the frontalis muscle in the distal portion and below the frontalis muscle in the proximal portion. An incision was extended across the orbital rim. In the first stage, flap insetting was performed. Three weeks later, the flap was divided, but a notching was observed at the alar margin of the reconstructed nose. One month after the later procedure, the inferior skin outer portion of the folded flap was incised along the future alar rim during an intermediate operation. The skin was elevated while preserving 2-3 mm of subcutaneous fat. Excess fatty tissue underlying the cartilage and inner lining tissue was removed to create a thin, pliable, and well-vascularized lining. The skin was sutured along the future alar margin. Then, three weeks later, the flap was divided, and the final insetting was performed [11]. 

**Figure 2:**
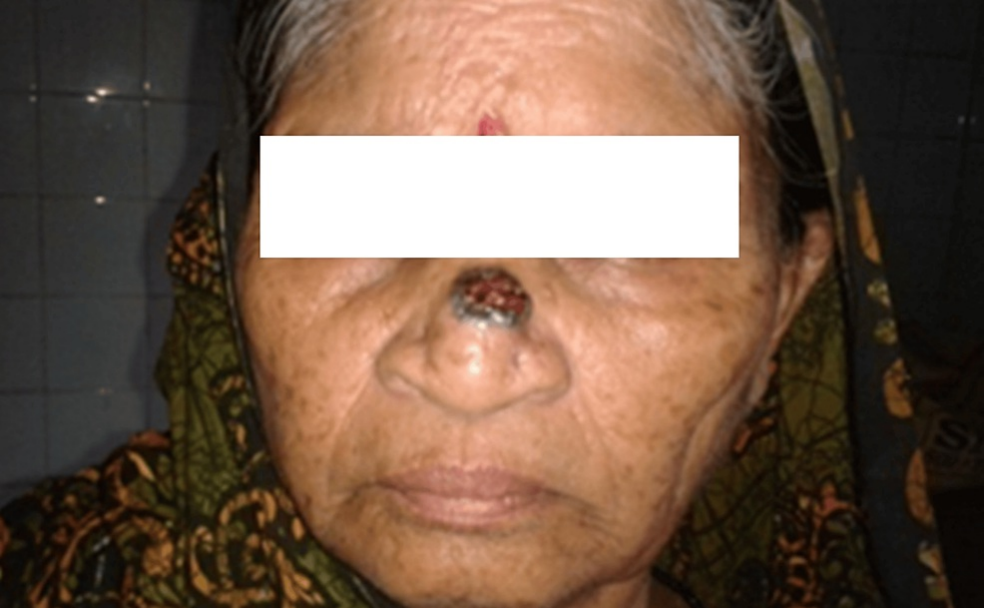
Preoperative view

The following images represent case no 3 (details provided later on in a table). Here, only one unit is involved, i.e., the nasal unit. If we further classify, a single subunit is also involved, i.e., only the dorsum of the nose is involved. This was reconstructed with a Rieger flap (Figures 1, 2).

**Figure 3:**
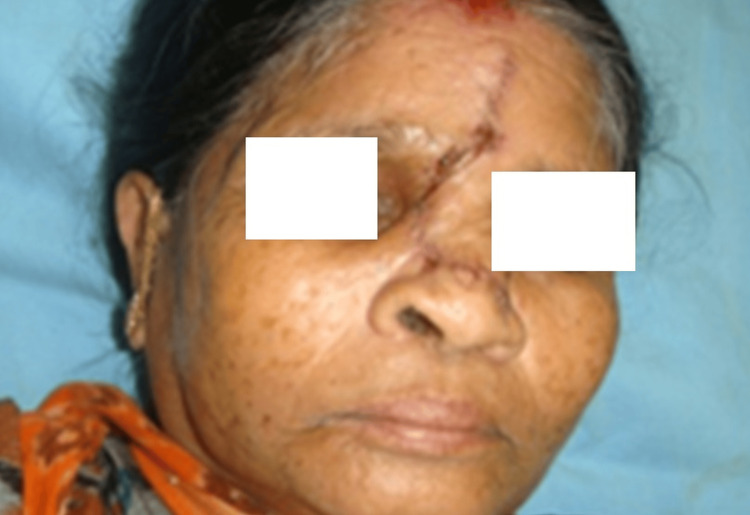
Postoperative view

The Mustardé flap is a sizable rotational cheek flap commonly employed for reconstructing lower eyelid defects. This flap transfers excess skin situated laterally to the lateral canthus and can be mobilized in an inferolateral direction to cover the lower eyelid region. The incision begins at the margin of the defect and proceeds outward and upward. If necessary, the incision can be extended downward in front of the auricle and then along the back of the auricle. To prevent the weight of the flap from causing downward retraction of the eyelid, the inner portion of the flap is secured to the periosteum of the zygoma, temporalis fascia, and canthus by using one or two permanent sutures of 5-0 prolene [11].

A cervical flap is particularly useful in middle-aged or older patients, capitalizing on the laxity of cervical skin and allowing for primary closure of the donor site. The incision extended along the lateral wall of the nose and nasolabial fold, further extending down to the mandible. During the flap elevation, care was taken to preserve the facial nerve by working above the superficial musculoaponeurotic system (SMAS) layer. The flap was anchored to the nose along its lateral wall and the zygoma to prevent ectropion. Meticulous hemostasis was maintained in all cases, and no drains were used. Closure of the wound was achieved with 5-0 prolene sutures [12].

Patients were followed up for a minimum of six months to a maximum of 12 months. To prevent scar hyperpigmentation, patients were advised to avoid sun exposure for six to nine months after surgery. Complications were observed in only two cases (5.7% of cases): in the form of mild congestion at the distal tip, which resolved within two weeks. There was no recurrence of the excised lesion.

## Results

The study comprised 35 patients, with 15 (42.85%) being males and 20 (57.15%) females, resulting in a male-to-female ratio of 1:1.33. Their ages ranged from 42 to 68 years. The demographic characteristics are shown in Table 1.

**Table 1:** Demographic characteristics FTSG: full-thickness skin graft; STSG: split-thickness skin graft

Sl no	Age, years	Sex	Site	Unit and subunits involved	Mode of reconstruction
1	48	F	Forehead	1a	Limberg flap
2	62	M	Forehead and cheek	1b, 4b	FTSG
3	52	F	Dorsum of nose	2a	Reiger flap
4	60	M	Dorsum and lateral wall of the nose	2a, 2b	Glabellar flap
5	65	M	Dorsum of nose	2a	Glabellar flap
6	63	M	Dorsum of the nose and medial canthus	2a, 3d	Glabellar flap
7	56	F	Lateral wall and ala of the nose	2b, 2c	Bilobed flap
8	48	M	Lateral wall, ala, and tip	2b, 2c, 2d	Forehead flap
9	42	F	Tip of nose	2d	Forehead flap
10	48	F	Tip of nose	2d	Nasolabial flap
11	59	F	Lateral wall, ala of nose, and cheek	2b, 2c, 4a	Forehead flap
12	56	F	Lateral cantus of eyelid	3c	Mustardé flap
13	45	F	Upper eyelid	3a	Cutler beard advancement flap
14	44	M	Lateral canthal region	3c	Limberg flap
15	61	F	Lower eyelid	3d	FTSG
16	45	F	Infraorbital cheek	4a	FTSG
17	52	M	Infraorbital cheek	4a	Limberg flap
18	44	F	Dorsum, lateral wall of the nose, medial canthus of the eye, and infraorbital cheek	2a, 2b, 3d, 4a	Forehead flap
19	64	M	Zygomatic, buccal area of the cheek	4b, 4d	Local transposition
20	61	F	The infraorbital area of the cheek and the lateral portion of the upper lip	4a, 5b	Nasolabial flap
21	56	M	Inferior eyelid, lateral canthus, and infraorbital area of the cheek	3b, 3c, 4A	Cervicofacial flap
22	49	F	infraorbital cheek, medial canthus of the eyelid, and inferior eyelid	3b, 3d, 4a	Mustardes flap
23	63	M	Infraorbital cheek	4a	Mustardes flap
24	49	F	infraorbital cheek, medial canthus of eyelid and inferior eyelid, lateral canthus eyelid	3b, 3d, 3c, 4a	Cervicofacial flap
25	50	F	Inferior eyelid and infraorbital cheek	3b, 4a	Cervicofacial flap
26	48	F	Dorsum, lateral wall of the nose, medial canthus, lower eyelid, and infraorbital cheek	2a, 2b, 3d, 3b, 4a	Paramedian forehead flap
27	46	F	Helical rim	8a	FTSG
28	59	M	Antihelix	8b	Composite graft
29	64	M	Helical rim and antihelix	8a, 8b	FTSG
30	59	M	Concha	8c	Flap
31	43	M	Retroauricular	8e	FTSG
32	49	F	Lip lateral wall and philtrum	5a, 5b	Nasolabial flap
33	57	F	Retroauricular	8e	Limberg
34	62	M	The lateral wall of the forehead and zygomatic area of the cheek	1b, 4b	STSG
35	64	F	Lateral canthus of the eyelid	3c	Mustardé flap

A significant majority of the patients (71.4%, n=25/35), hailed from rural areas, with the majority being illiterate. Of note, we observed an association between the duration of the disease and literacy. The lack of awareness among illiterate patients often leads to delayed medical presentations. The duration of the disease before seeking medical care ranged from one year to nine years.

Among the female patients, there was intermittent exposure to high-intensity ultraviolet radiation (UVR), and none of the patients applied sunscreen on their faces to mitigate the hazardous effects of prolonged UVR. Additionally, there was no history of treatment with psoralen plus ultraviolet A (PUVA). All female patients were non-alcoholics and non-smokers. Among the male patients, 60% were smokers, and 20% both smoked and consumed alcohol. All patients belonged to Fitzpatrick skin types 3 and 4.

Remarkably, all flaps employed in the procedures survived, and there were no instances of flap necrosis. Patients were followed up for a minimum of one year, with no instances of recurrence during the follow-up period ranging from six to 18 months. Furthermore, there were no cases of wound dehiscence, wound hematoma, or seroma.

## Discussion

BCC exhibits a higher prevalence among males worldwide, likely due to greater UVR exposure [12-15]. Surprisingly, our study found a higher incidence among females, in line with some other studies. This discrepancy could be attributed to the fact that Indian housewives, especially rural women, are exposed to intermittent, intense UVR during household chores and fieldwork [14-18]. BCC primarily results from cumulative UVR exposure. While research by Mancuso et al. suggested a potential role of sex hormones such as estrogen in skin cancer development, this relationship remains understudied [19]. Skin malignancies are most commonly found on the face, typically in older individuals, but BCC can also occur in younger age groups [20]. However, in our study, all patients were over 40 years old.

Notably, people with dark skin usually show signs of BCC later on, because telangiectasia and the raised pearly border, which are signs of BCC, are harder to see in them. Pigmented tumors are also more frequent in individuals with darker skin, with a 44.2% incidence rate compared to an average of 5% in the Caucasian population [21, 22]. The nose, eyelid, and cheek were the most frequently affected sites in our series regarding BCC involvement. This contrasts with some other studies reporting the lip, orbit, and nasolabial region as the more common sites [23-27]. Specifically, we found that the root of the nose and the proximal third were commonly involved. In nose reconstruction, skin grafts can result in a shiny, atrophic appearance, while flaps offer better color and texture match. The flap options include local transposition flaps, rotation flaps, advancement flaps, forehead flaps, nasolabial flaps, and radial artery forearm flaps [28].

In our cases, we used various flaps, such as the glabellar flap, dorsal nasal flap, and bilobed flap, for superficial defects up to three cm. However, full-thickness nose defects were typically reconstructed with the folded forehead flap. We did not use the free radial forearm flap. The guidelines for nose reconstruction were based on defect size and location, with options like single or double transposition flaps and bilobed flaps [28]. The most common axial pattern flap used in our series was the forehead flap, which is a versatile option for subtotal and total nasal defects. The nasolabial flap was the second most common choice, suitable for sidewall, ala, and tip defects. All eyelid BCC cases in our study involved more than one zone and had extensive lesions. Most eyelid reconstructions used the Mustardé and cervicofacial flaps. Surgical resection proved to be better than Mohs micrographic surgery. For smaller defects, local flaps were used, but for larger periocular defects, cervicofacial and Mustardé flaps were required. Medial canthal defects with nasal involvement were addressed using the glabellar flap.

In cheek reconstruction, consideration of anatomical subunits was crucial for flap selection, aiming for better color and texture match. Local flaps were favored, with meticulous planning to avoid contour deformities in adjacent structures. The Limberg flap was commonly used for isolated cheek lesions. Reconstruction of the lateral wall, tip, and dorsum of the nose required various flaps due to the anatomical complexity of these regions. Flap choices in our series align with previous findings. The dorsum of the nose was often reconstructed using the Rieger flap and the glabellar flap. Limberg and A-T flaps were not used in our cases. The Limberg flap and lateral advancement flap were used for lateral wall reconstruction, although we used different flaps for larger, multi-subunit defects.

The Limberg flap was the most common choice for reconstructing defects along the lateral canthus of the eye, similar to previous findings. Mustardé and cervicofacial flaps were commonly used for infraorbital cheek areas in our series, consistent with observations by Russo et al. [29]. Our study was the second of its kind to describe lesions based on involvement in multiple units and subunits. Van Leeuwen et al.'s study indicated that single aesthetic units were involved in 79.7% of cases, while multiple units were involved in 20.3% [30]. In our study, single units were involved in 68.57% of cases, major double units in 25.71%, and three major units in 5.71%.

Our reconstruction approaches are aligned with Van Leeuwen et al.'s, except for using an island nasolabial flap for lip reconstruction instead of the Abbe flap. Damage to the forehead was fixed with rotation advancement flaps, damage to the area around the eyes with cheek rotation flaps, damage to the nose with forehead flaps followed by bilobed flaps and dorsal nasal flaps, damage to the cheeks with rotation advancement flaps, and damage to the upper lip with the Abbe flap [30]. We found that cervicofacial flaps were useful for defects in the lower eyelid and infraorbital cheek areas, while Mustardé flaps were more suitable for laterally placed defects. As defects extended medially, Mustardé flaps sometimes required a skin graft, diminishing their aesthetic value. In contrast, cervicofacial flaps allowed for primary closure, resulting in better cosmetic outcomes [31]. In summary, our study sheds light on the gender and ethnic variations in BCC prevalence. Reconstruction methods were chosen based on anatomical considerations and the reconstructive ladder approach for optimal cosmetic results. Scar placement along relaxed skin tension lines (RSTL) was essential for achieving favorable outcomes.

The study has a few limitations, including its relatively small sample size and potential data bias. We recommend further multicenter randomized trials to determine flap superiority in a conclusive manner [32-34].

## Conclusions

Our study highlights the importance of adhering to the reconstructive ladder in the management of BCC to achieve optimal outcomes. Small defects resulting from BCC excision can be effectively reconstructed using local flaps, while larger lesions involving multiple subunits may necessitate the use of locoregional flaps. Careful planning of the reconstructive procedure is crucial to ensure that the final scar is positioned along the junction of facial subunits, thereby optimizing aesthetic outcomes. By following these principles, surgeons can achieve successful reconstruction while preserving facial aesthetics and functionality in patients with BCC.
